# Association between abdominal muscle mass measured by dual-source computed tomography and coronary artery calcification in patients with Type 2 diabetes mellitus

**DOI:** 10.3389/fendo.2026.1803735

**Published:** 2026-03-26

**Authors:** Xiaolong Chen, Meidi Tan, Limin Liu, Lu Ye, Chunyu Wu, Xihua Zhou, Wenxuan Lei, Mingxuan Huang, Fei Peng

**Affiliations:** 1 Department of Radiology, The First Affiliated Hospital, Hengyang Medical School, University of South China, Hengyang, China; 2 Department of Ophthalmology, The First Affiliated Hospital, Hengyang Medical School, University of South China, Hengyang, China; 3 Department of Ultrasound, The Second Affiliated Hospital, Hengyang Medical School, University of South China, Hengyang, Hunan, China; 4 Department of Radiology, Changde Municipal First Hospital of Traditional Chinese Medicine, , Changde, Hunan, China

**Keywords:** abdominal muscle mass, coronary artery calcification, dual-source computed tomography, muscle mass index, type 2 diabetes mellitus

## Abstract

**Objective:**

To describe the correlation between abdominal muscle mass and CAC in T2DM patients using DSCT, and to determine the preferred muscle imaging indicators in diagnosing and predicting positive CAC patients.

**Materials and methods:**

108 T2DM patients were included (57.0 ± 10.9 years old). We acquired both CAC score and abdominal-chest DSCT data. DSCT measurements (intermuscular adipose tissue [IMAT], total abdominal muscle area [TAMA], normal attenuation muscle area [NAMA], low attenuation muscle area [LAMA], fat fraction [FF], LAMA/body mass index [BMI], NAMA/BMI, and NAMA/TAMA index) of 7 muscles (psoas major [PM], quadratus lumborum [QL], erector spinae [ES], rectus abdominis [RA], transversus abdominis [TA], oblique abdominals [OA], abdominal core muscles [ACM]) on the level of the third lumbar vertebra were conducted. T2DM patients were divided into four subgroups based on CAC score: negative controls (NCs) (0 score), mild (<100 score), moderate (100–300 score), and severe (>300 score). The following statistical analyses were conducted: intergroup differences were compared using the Mann-Whitney test, diagnostic performance was evaluated via receiver operating characteristic (ROC) curve analysis, associations were assessed with Spearman correlation, and predictors were identified through logistic regression. A *P*-value <0.05 was considered statistically significant.

**Result:**

Compared with NCs, TAMA, NAMA, NAMA/BMI, and NAMA/TAMA index were significantly higher in the group of all CAC (*P*<0.05), while TAMA was significantly lower (*P*<0.05). The NAMA, NAMA/BMI and the NAMA/TAMA index in four representative muscles (PM, QL, ES, and RA) demonstrated certain diagnostic performance (AUC range, 0.70-0.97, 0.85-0.99 and 0.81-0.97). Overall, NAMA/TAMA index in OA exhibited the strongest negative correlation with CAC score (r=-0.56, *P* < 0.01), and NAMA/BMI in the ACM emerged as an independent risk factor for positive CAC (odds ratio=0.63, *P* = 0.02).

**Conclusion:**

Abdominal muscle mass measured by DSCT was significantly associated with CAC score in T2DM patients. Overall, the NAMA/BMI showed optimal diagnostic value for CAC across severity levels. Moreover, NAMA/BMI in the ACM may serve as a predictive biomarker for positive CAC.

## 1 Introduction

Diabetes mellitus (DM) is a globally prevalent metabolic disease, with type 2 diabetes mellitus (T2DM) being the predominant form (1, 2). Despite advances in diagnosis and treatment, T2DM patients still bear a high cardiovascular burden (3, 4). In this population, coronary artery disease (CAD) is the leading cause of death and often progresses silently to life-threatening events without obvious symptoms (2). Atherosclerosis drives most CAD cases and is closely tied to coronary artery calcification (CAC) (3, 5, 6). Thus, CAD screening and detection in T2DM is vital for timely intervention and preventing progression, highlighting an emerging public health priority.

At present, identification of high cardiovascular risk in patients with T2DM remains challenging. Current approaches rely mainly on laboratory biochemical markers and traditional risk scores, yet these methods have limitations in capturing heterogeneous risk profiles against a background of metabolic dysregulation (2, 7). Therefore, there is an urgent need in clinical practice to develop reliable CAC biomarkers for patients with T2DM. Recent studies have shown significant associations between abdominal muscle mass and calcification of the aorta and carotid arteries, suggesting that abdominal muscle mass may also participate in the pathological process of cardiovascular calcification in patients with T2DM (8, 9). Accordingly, we propose that abdominal muscle mass is likely associated with cardiovascular calcification in this population and could serve as an important complementary means for identifying high cardiovascular risk in T2DM patients.

In recent years, computed tomography (CT) has become an important tool for evaluating abdominal muscle mass, including skeletal muscle structure, composition, and fat infiltration (7). Among these techniques, dual-source computed tomography (DSCT) enables precise differentiation of muscle and fat components by applying material decomposition algorithms at different energy levels, without incurring additional radiation exposure or contrast medium, and allows seamless integration of multidimensional muscle parameter measurements into routine thoracoabdominal CT examination workflows (6, 10, 11). Based on the potential association between abdominal muscle mass and cardiovascular calcification, we speculate that DSCT-derived abdominal muscle measurements may provide reliable outcomes and aid in risk stratification for T2DM population.

The aim of this study was to investigate the relationship between DSCT-derived abdominal muscle measurements and CACS in patients with T2DM. We also aimed to determine the ability of muscle parameters to identify severity of CAC. Lastly, we further identified the potential of muscle parameters as a predictive tool for CAC.

## 2 Methods

### 2.1 Study population

This observational study was approved by the Institutional Review Board, and the clinical trial registration was completed (registration number: No. 2021LL0426001). Written informed consent was obtained from all parents before participating in the study.

**Figure 1:**
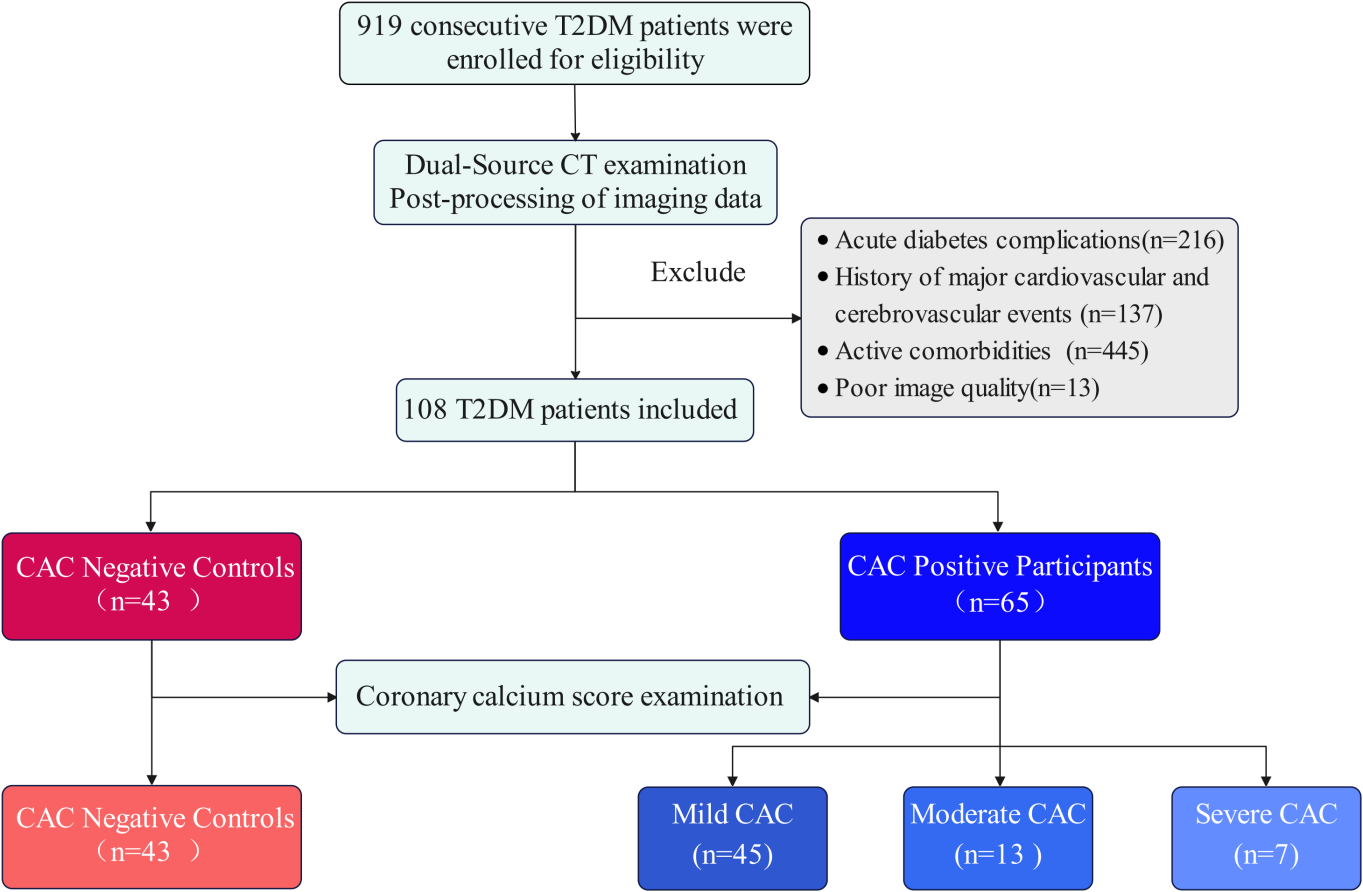
Flowchart showing inclusion and exclusion criteria for the study population. T2DM, type 2 diabetes mellitus; CAC, coronary artery calcification; CT, Computed Tomography.

Patients with T2DM admitted to the Department of Endocrinology, First Affiliated Hospital of University of South China, will be prospectively enrolled between January 2021 and December 2023. All participants will undergo standardized assessments including: (1) DSCT of the abdomen and chest, (2) comprehensive biochemical and immunological profiling, (3) cognitive function evaluation using validated instruments. Inclusion criteria for T2DM participants will be diagnosed on the basis of biochemical and immunological testing. Exclusion criteria comprise: a) acute diabetes complications (e.g., secondary or monogenic diabetes), b) history of major cardiovascular/cerebrovascular events (myocardial infarction, stroke, heart failure, or coronary interventions), or c) active comorbidities including malignancies, endocrine disorders (thyroid/parathyroid dysfunction), autoimmune diseases, severe infections, or disabling musculoskeletal conditions. Demographic, clinical, and laboratory data will be prospectively extracted from Hospital Information System (Jiahemaikang Inpatient EMR System Version 6.0, Beijing Jiahemaikang Information Technology Co., Ltd., China). **Figure 1** illustrates the patient selection and exclusion process.

### 2.2 Definition of diabetes

The diagnostic criteria for diabetes were as follows: the presence of diabetes symptoms, along with a random blood glucose concentration of 11.1 mmol/L or higher, a fasting plasma glucose concentration of 7.0 mmol/L or higher, or a blood glucose concentration of 11.1 mmol/L or higher two hours after a meal. In accordance with the international guidelines for the prevention and control of T2DM, the assessment of diabetes complications encompassed diabetic nephropathy, peripheral neuropathy, retinopathy, and lower-extremity arterial disease (12, 13).

### 2.3 CT scanning protocol

Abdominal and chest CT scans were conducted using a dual-source Revolution CT scanner (General Electric Medical Group, USA). The scan encompassed the chest from the lung apices to the costophrenic angles and the abdomen from the dome of the diaphragm to the symphysis pubis. For the abdominal scans, non - contrast dual - energy CT scans were acquired at tube voltages of 80 kVp and Sn140 kVp. The tube current was modulated, with a reference setting of 100 mAs for each tube to guarantee an adequate signal - to - noise ratio. The acquired dual - energy data sets were processed to generate simulated single - energy CT images. This was achieved by calculating a weighted average of the two image sets, applying a mixing ratio of 0.4, and using a soft -tissue reconstruction kernel in conjunction with sinogram-affirmed iterative reconstruction. The resultant images had a slice thickness of 1.25 mm and an interval of 1.0 mm.

### 2.4 Image analysis

Two radiologists with 3 and 6 years of experience in musculoskeletal CT imaging, respectively, delineated target areas on CT images. We used abdominal axial CT images at the third-lumbar (L3) level and the sliceOmatic version 5.0 Rev-9 (TomoVision, Magog, Canada) image analysis software based on the established Hounsfield unit (HU) threshold for muscle tissue to assess the body composition of participants. Following confirmation of inter-observer consistency, we used the average of the two radiologists’ region of interest (ROI) measurements as the final value for each muscle.

#### 2.4.1 Measurement of CAC

The images were reconstructed at a slice thickness of 3.0 mm with a medium-smooth convolution kernel. A dedicated field of view (FOV) of 250 mm encompassing the heart was used for analysis. All images were transferred to a dedicated workstation (Advantage AW 4.7 Workstation, General Electric, USA), and CACS was performed using SmartScore software (version 4.0, General Electric, USA). Lesions with a density of ≥130 Hounsfield units (HU) and a minimum area of 1 mm² were identified as calcifications. The Agatston score, which incorporates both the area and peak density of calcified lesions, was automatically calculated by the software for each coronary artery (left main, left anterior descending, left circumflex, and right coronary artery). The total CACS was calculated by summing the scores from all coronary arteries. The severity of CAC was graded based on the CACS: 0, negative; <100 score, mild; 100–300 score, moderate; >300 score, severe (12).

#### 2.4.2 Measurement of muscle mass

**Figure 2:**
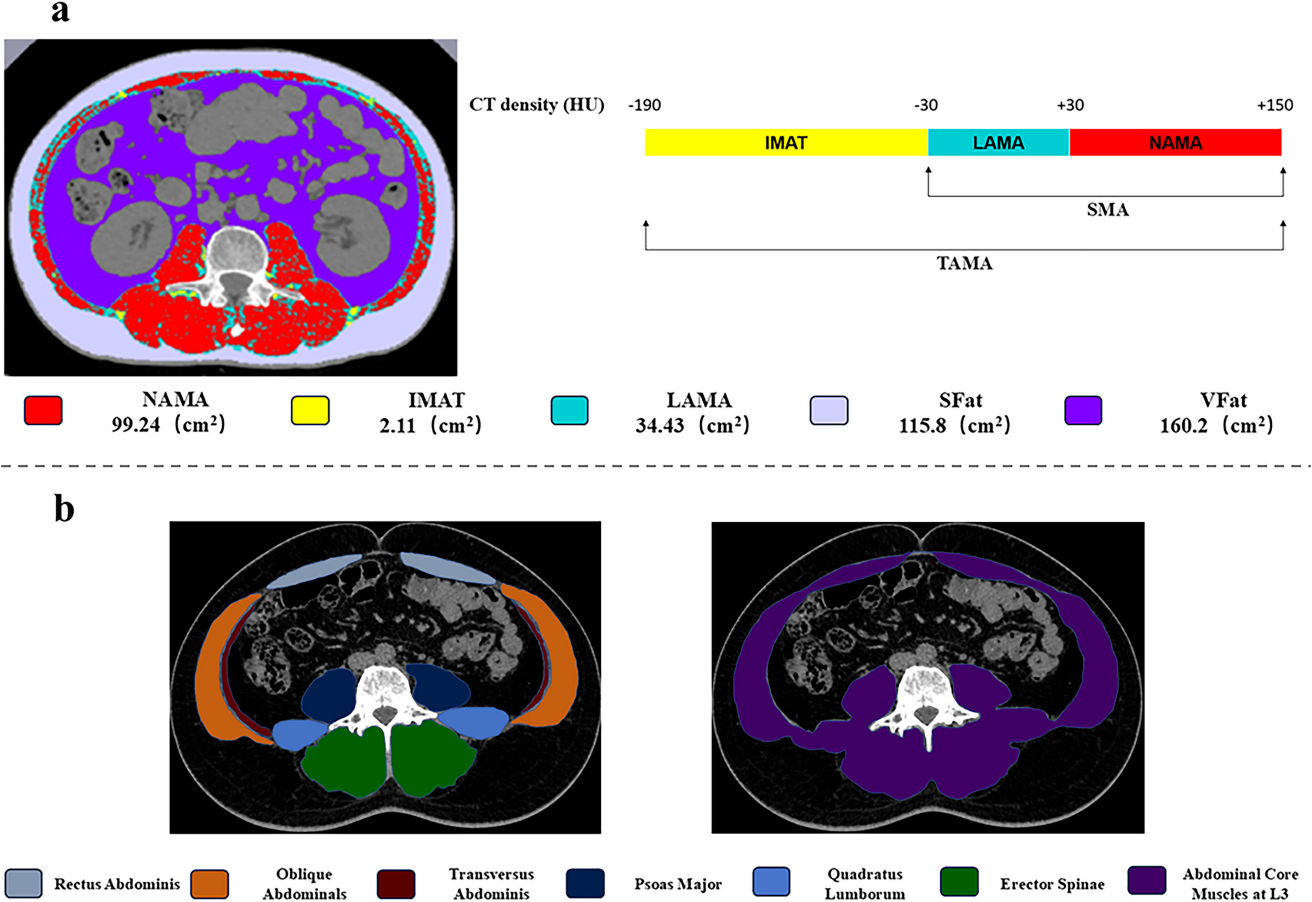
**(a)** Abdominal muscle threshold segmentation map of T2DW patients body composition analysis derived from an axial CT slice at the L3 level; **(b)** ROI selection for abdominal muscles. TAMA, total abdominal muscle area; SMA, skeletal muscle area; IMAT, intramuscular adipose tissue; LAMA, low attenuation muscle area; NAMA, normal attenuation muscle area; SFat, subcutaneous fat area; VFat, visceral fat area.

Seven abdominal muscles at the L3 level were selected for CT measurements. These muscles included psoas major (PM), quadratus lumborum (QL), erector spinae (ES), rectus abdominis (RA), transversus abdominis (TA), oblique abdominals (OA), abdominal core muscles (ACM). During the process of muscle target area delineation, the ROI was required to encompass the muscle as much as possible without extending beyond its margins. In CT scans, the attenuation range for NAMA (normal attenuation muscle area) was from +30 to +150 HU, the attenuation range for LAMA (low attenuation muscle area) was from -29 to +29 HU, the attenuation range for SAMA (skeletal muscle area) was from -29 to 150 HU, the attenuation range for VFA (visceral fat area) was from -150 to -30 HU, the attenuation range for SFat (subcutaneous fat area) was from -190 to -30 HU, and the attenuation range for IMAT (intramuscular adipose tissue) was from -190 to -30 HU (**Figure 2**). Fat fraction (FF), which serves as a quantitative imaging biomarker for assessing muscle fatty infiltration, was then calculated. FF was derived using the following formula: FF (%) = LAMA/TAMA] × 100%.

### 2.5 Statistical analysis

The normality of the data distribution was assessed using the Shapiro-Wilk test. A Mann-Whitney U test was used to compare demographic characteristics and CT measurements between all CAC positive participants and NCs (negative controls), performed, as well as for comparisons encompassing mild CAC, moderate CAC, severe CAC, and NCs. To mitigate Type I error, the statistical significance threshold for the Mann-Whitney U test was adjusted using the Benjamini-Hochberg correction. Receiver operating characteristic (ROC) analysis, based on the area under the curve (AUC), was performed to determine the muscle measurements most effective in distinguishing between CAC subgroups and NCs. Additionally, Spearman rank correlation analysis was employed to assess the correlation between muscle measurements and CACS. To identify independent predictive factors for CAC positive patients with T2DM, logistic regression analysis was performed. Based on the literature (14, 15), the multivariable logistic regression analysis was further adjusted for the following variables: age, sex, low-density lipoprotein (LDL), high-density lipoprotein (HDL), triglycerides (TG), glycated hemoglobin (HbA1c, %), duration of diabetes (months), family history of CVD (n, %), osteoporosis (n, %), hypertension (n, %), alcohol history (n, %), and smoking history (n, %). All statistical analyzes were conducted using SPSS version 27.0, MedCalc version 15.2.0, and GraphPad Prism version 10.5.0. A *P*-value<0.05 was considered to indicate statistical significance.

## 3 Results

### 3.1 Participant characteristics

After excluding 13 patients due to poor image quality, 216 with acute diabetes complications, 137 with a history of major cardiovascular and cerebrovascular events, and 445 with active comorbidities, a total of 108 T2DM patients were finally included. According to CACS, T2DM patients were categorized four subgroups: 43 NCs (55.5 ± 12.3 years), 45 with mild CAC (57.6 ± 9.8 years), 13 with moderate CAC (57.6 ± 8.5 years), and 7 with severe CAC (66.7 ± 5.9 years) (**Figure 1****).**

The demographic and clinical characteristics of the participants are shown in **Table 1**. This study included 65 CAC-positive patients and 43 NCs. There were no significant differences in age, height, weight, osteoporosis, hypertension, alcohol or smoking history, HDL cholesterol, triglycerides, body mass index (BMI), HbA1c between the CAC-positive subgroup and NCs (all *P*>0.05). Compared to NCs, the CAC-positive participants had a significantly higher prevalence of family history of cardiovascular disease, higher LDL cholesterol levels, longer duration of diabetes (all *P* < 0.05).

**Table 1:** Demographics of CAC positive participants and CAC negative controls with T2DM.

Variable	CAC negative controls (n=43)	CAC positive participants (n=65)	*P*-value
Age (years)	55.0 ± 12.0	59.0 ± 10.0	0.051
Height (cm)	161.7 ± 8.5	159.9 ± 8.0	0.274
Weight (kg)	63.8 ± 11.9	61.2 ± 11.3	0.262
BMI	24.3 ± 3.4	23.8 ± 3.5	0.541
HbA1c (%)	10.4 (9.0-12.8)	9.8 (8.3-11.6)	0.227
Diabetes Duration (months)	24 (1-96)	84 (36-120)	<0.001
Family history of CVD (n, %)	0 (0)	50 (76.9)	<0.001
Osteoporosis (n, %)	31 (72.1)	51 (78.5)	0.451
Hypertension (n, %)	12 (27.9)	18 (27.7)	0.981
Alcohol history (n, %)	4 (10.0)	6 (9.2)	0.897
Smoking history (n, %)	8 (18.6)	8 (12.3)	0.369
TG (mmol/L)	1.55(1.2-2.4)	1.59(1.1-2.6)	0.903
LDL-C (mmol/L)	2.42(1.8-2.9)	2.75(2.3-3.1)	<0.05
HDL-C (mmol/L)	0.95(0.8-1.1)	0.97(0.8-1.1)	0.940

Values are mean (SD), median (interquartile ranges), or number (%). CAC, coronary artery calcification, BMI, body mass index; CVD, cardiovascular disease; HbA1c, hemoglobin A1c; TG, Triglycerides; LDL-C, low-density lipoprotein cholesterol; HDL-C, high-density lipoprotein cholesterol.

### 3.2 Muscle measurements in CAC positive participants and NCs with T2DM

**Table 2** presents the DSCT measurements (IMAT, TAMA, NAMA, LAMA, FF, LAMA/BMI, NAMA/BMI, and NAMA/TAMA index) of 7 muscles among T2DM patients. A significant difference was observed in TAMA, NAMA, NAMA/BMI, and the NAMA/TAMA index between the NCs and mild CAC, moderate CAC, and severe CAC groups (*P* < 0.05). By comparison, no significant differences were observed in IMAT, LAMA, FF, LAMA/BMI (*P* > 0.05) (**Supplementary Table 1**).

**Table 2:** DSCT measurements of individual muscles among NCs CAC, Mild CAC, Moderate CAC, and Severe CAC participants.

Muscle	TAMA (cm^2^)						NAMA (cm^2^)						NAMA/BMI (cm^2^/kg/m^2^)						NAMA/TAMA index (%)					
	NCs CAC (n=43)	Mild CAC (n=45)	Moderate CAC (n=13)	Severe CAC (n=7)	All CAC (n=65)	*P* for trend	NCs CAC (n=43)	Mild CAC (n=45)	Moderate CAC (n=13)	Severe CAC (n=7)	All CAC (n=65)	*P* for trend	NCs CAC (n=43)	Mild CAC (n=45)	Moderate CAC (n=13)	Severe CAC (n=7)	All CAC (n=65)	*P* for trend	NCs CAC (n=43)	Mild CAC (n=45)	Moderate CAC (n=13)	Severe CAC (n=7)	All CAC (n=65)	*P* for trend
PM	19.12 ± 0.96	20.37 ± 0.95^c^	14.80 ± 1.45^b^	15.80 ± 2.27^a^	20.37 ± 0.95^d^	<0.05	13.90 ± 0.84	14.84 ± 0.79^c^	10.88 ± 1.28^b^	9.39 ± 1.89^a^	13.32 ± 2.25^d^	<0.05	5.71 ± 0.32	6.03 ± 0.28^c^	4.9 ± 0.61^b^	4.1 ± 0.76^a^	5.54 ± 0.79^d^	<0.05	71.70 ± 1.78	72.44 ± 1.65^c^	72.97 ± 3.79^b^	59.10 ± 6.85^a^	70.53 ± 4.76^d^	<0.05
QL	10.73 ± 0.51	9.82 ± 0.45	8.39 ± 0.59^b^	8.16 ± 1.34	9.36 ± 0.93^d^	<0.05	8.07 ± 0.44	7.28 ± 0.38^c^	5.84 ± 0.52^b^	4.70 ± 1.20^a^	6.64 ± 1.02^d^	<0.05	3.31 ± 0.16	2.96 ± 0.13^c^	2.63 ± 0.23^b^	1.96 ± 0.44^a^	2.76 ± 0.33^d^	<0.05	71.97 ± 1.81	71.85 ± 1.51^c^	69.82 ± 4.41^b^	54.91 ± 7.40^a^	68.79 ± 5.85^d^	<0.05
ES	48.63 ± 1.54	48.00 ± 1.50	43.41 ± 2.52	44.86 ± 3.88^a^	46.74 ± 2.81^d^	0.164	34.17 ± 1.64	33.33 ± 1.62^c^	27.78 ± 2.75^b^	22.17 ± 3.6^a^	31.35 ± 3.78^d^	<0.05	14.11 ± 0.62	13.75 ± 0.65^c^	12.69 ± 1.31^b^	9.43 ± 1.09^a^	13.05 ± 1.62^d^	<0.05	70.07 ± 2.59	69.48 ± 2.85^c^	63.62 ± 4.89^b^	48.39 ± 5.42^a^	65.93 ± 6.33^d^	<0.05
RA	24.98 ± 1.18	26.20 ± 1.21	19.51 ± 1.66^b^	19.27 ± 2.36	24.12 ± 3.46^d^	<0.05	14.04 ± 0.84	14.83 ± 0.81^c^	10.47 ± 1.19^b^	10.84 ± 1.70^a^	13.77 ± 2.21^d^	<0.05	5.79 ± 0.33	6.03 ± 0.30^c^	4.72 ± 0.57^b^	4.86 ± 0.77^a^	5.67 ± 0.67^d^	<0.05	54.91 ± 1.25	55.65 ± 1.16^c^	52.24 ± 2.45^b^	54.54 ± 3.40^a^	55.26 ± 2.34^d^	<0.05
TA	7.44 ± 1.16	10.89 ± 0.45	11.14 ± 0.50^b^	8.76 ± 0.70^a^	10.71 ± 0.84^d^	<0.05	6.32 ± 0.58	6.70 ± 0.55	4.25 ± 0.85	2.69 ± 1.14^a^	5.86 ± 1.53^d^	<0.05	2.63 ± 0.24	2.73 ± 0.22^c^	1.91 ± 0.39^b^	1.16 ± 0.48^a^	2.48 ± 0.49^d^	<0.05	55.20 ± 3.82	56.86 ± 3.38	44.76 ± 6.77	28.56 ± 10.88^a^	52.07 ± 8.39^d^	<0.05
OA	46.20 ± 1.83	45.32 ± 1.72	37.49 ± 2.89	39.29 ± 3.70	43.10 ± 4.04^d^	<0.05	29.68 ± 1.73	29.78 ± 1.40^c^	21.45 ± 2.65^b^	17.67 ± 3.61^a^	27.09 ± 4.43^d^	<0.05	12.25 ± 0.69	12.2 ± 0.53^c^	9.68 ± 1.19^b^	7.63 ± 1.38^a^	11.48 ± 1.31^d^	<0.05	63.19 ± 2.31	65.44 ± 1.98^c^	56.77 ± 5.18^b^	44.00 ± 6.23^a^	62.08 ± 6.06^d^	<0.05
ACM	140.9 ± 4.6	141.8 ± 4.2	118.3 ± 7.2^b^	123.1 ± 11.4	135.1 ± 11.8^d^	<0.05	91.23 ± 4.72	92.68 ± 4.06^c^	69.96 ± 6.80^b^	68.86 ± 10.54^a^	86.94 ± 10.97^d^	<0.05	37.78 ± 1.87	38.05 ± 1.56^c^	31.65 ± 3.16^b^	30.62 ± 4.95^a^	36.32 ± 3.92^d^	<0.05	63.78 ± 2.07	64.46 ± 1.74^c^	58.16 ± 3.16^b^	54.38 ± 5.27^a^	62.08 ± 4.39^d^	<0.05

Data represent mean± SD. An adjustment in statistical significance thresholds has been implemented by using Benjamini-Hochberg correction.

CAC, coronary artery calcification; NCs, CAC Negative Controls; PM, psoas major; QL, quadratus lumborum; ES, erector spinae; RA, rectus abdominis; TA, transversus abdominis; OA, oblique abdominals; ACM, abdominal core muscles at L3.

NAMA = normal attenuation muscle area, TAMA= total abdominal muscle area, BMI= body mass index, NAMA/TAMA index= normal attenuation muscle area/total abdominal muscle area×100.

a *P* < 0.05, Severe CAC vs. NCs.

b *P* < 0.05, Moderate CAC vs. NCs.

c *P* < 0.05, Mild CAC vs. NCs.

d *P* < 0.05, All CAC vs. NCs.

Across the T2DM subgroups with NCs, mild CAC, moderate CAC, severe CAC and all CAC, the mean TAMA values ranged from 7.44–140.9cm², 9.82–141.8cm², 8.39–118.3cm², 8.76–123.1cm², and 9.36–135.1cm², respectively. The corresponding mean NAMA values were 6.32–91.23cm², 6.70–92.68cm², 4.25–69.96cm², 2.69–68.86cm² and 6.64–86.94cm², while the mean NAMA/BMI measurements were 2.63–37.78, 2.73–38.05, 1.91–31.65, 1.16–30.62 and 2.48–36.32 cm²/kg/m², respectively. The mean NAMA/TAMA index values were 54.91%–71.97%, 55.65%–72.44%, 44.76%–72.97%, 28.56%–59.10% and 52.07%–70.53%, respectively.

**Figure 3:**
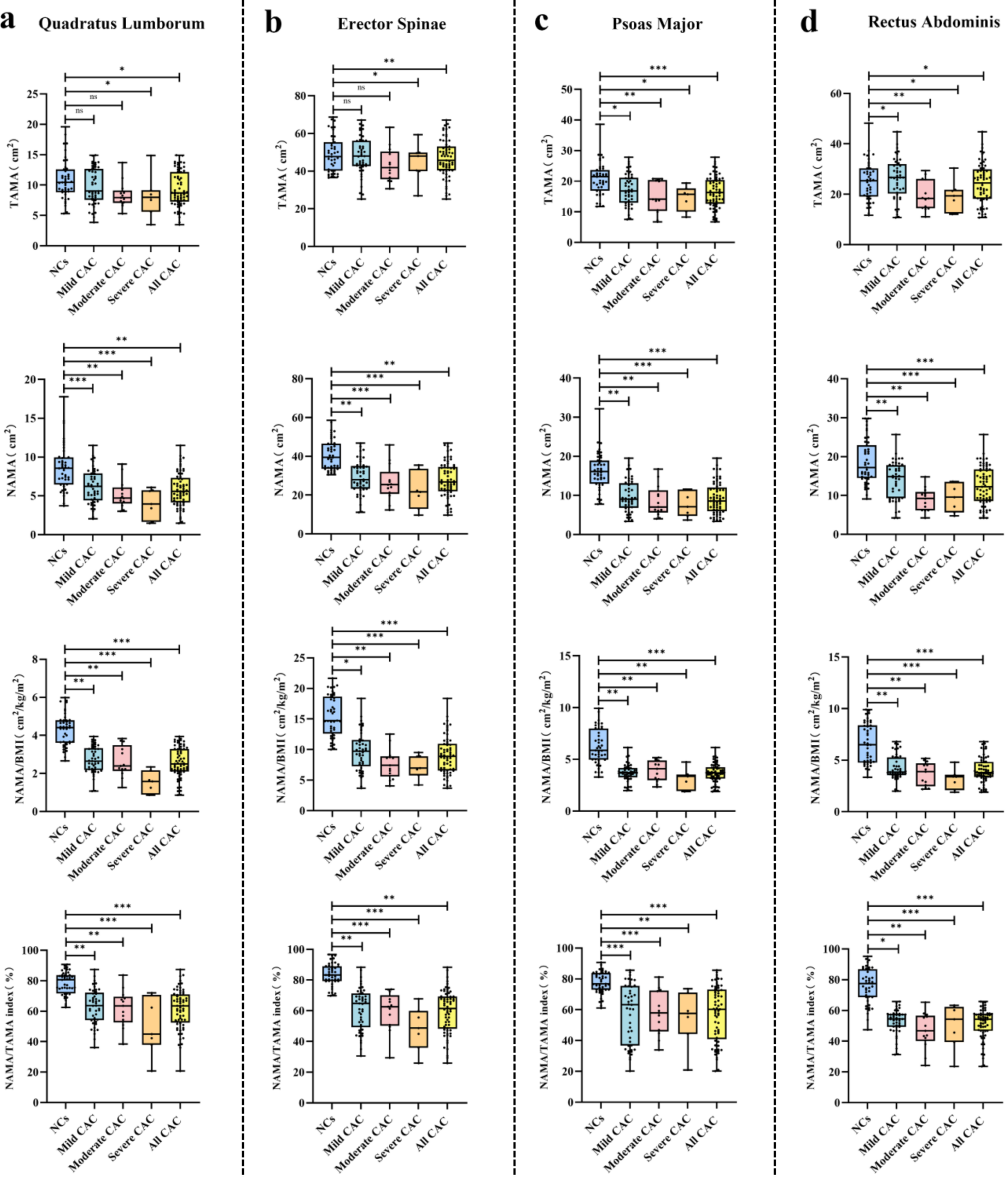
Boxplots of DSCT measurement biomarker [TAMA, NAMA, NAMA/BMI, and NAMA/TAMA index) for four representative muscles (quadratus lumborum **(a)**, erector spinae **(b)**, psoas major **(c)** and rectus abdominis **(d)**] that were used for comparison among CAC Negative Controls (NCs), mild CAC, moderate CAC, severe CAC, and All CAC participants. Statistically significant, *P < 0.05, **P < 0.01, ***P < 0.001. CAC, coronary artery calcification; NAMA, normal attenuation muscle area; TAMA, total abdominal muscle area; BMI, body mass index, NAMA/TAMA index, normal attenuation muscle area/total abdominal muscle area×100; NS, no significance.

Compared to the NCs, all 7 muscles exhibited significantly higher NAMA, NAMA/BMI, and the NAMA/TAMA index in mild CAC, moderate CAC, and severe CAC groups. the Mild CAC group exhibited significantly greater TAMA in the PM and TA, as well as higher NAMA in the PM, TA, and ACM (*P* < 0.05). In contrast, the moderate and severe CAC subgroup showed significantly lower TAMA in the PM and ACM, along with reduced NAMA in the ES and ACM (*P* < 0.05). Additionally, the NAMA/TAMA index was significantly decreased in the severe CAC subgroup for the PM, ES, and ACM (*P* < 0.05). **Figure 3** presents the DSCT measurements in selected representative muscles (QL, ES, PM, RA) across the study subgroups: NCs, and mild, moderate, severe, and all CAC subjects.

### 3.3 Diagnostic performance of muscle measurements for CAC positive participants with T2DM

**Figure 4:**
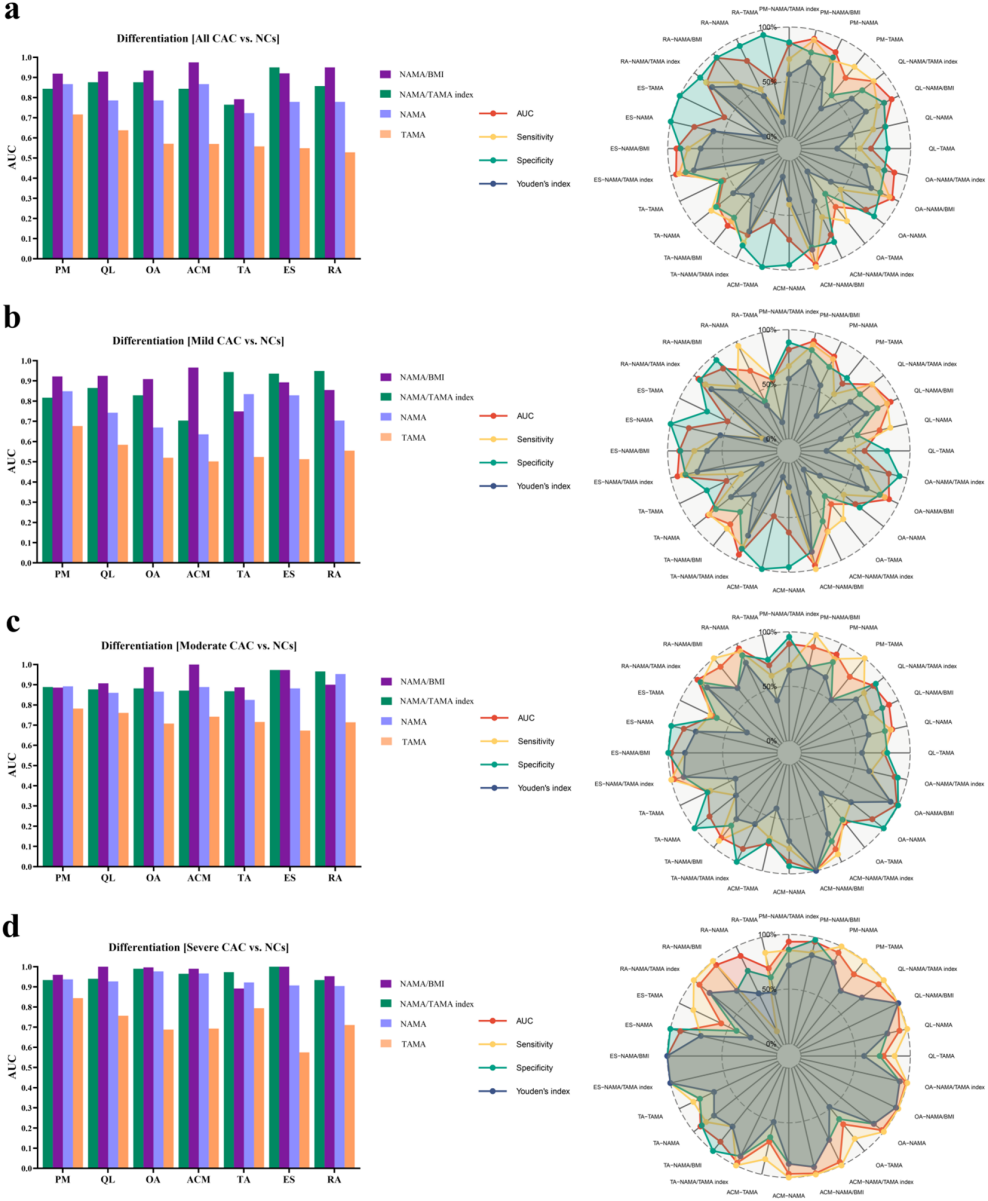
Area under the curve (AUC) value and radar chart of four DSCT measurements (TAMA, NAMA, NAMA/BMI, and NAMA/TAMA index) in individual muscles to differentiate between all CAC with T2DM and CAC negative controls (NCs) **(a)**, as well as between CAC-positive subgroups (mild, moderate, and severe) and NCs **(b-d)**. PM, psoas major; QL, quadratus lumborum; ES, erector spinae; RA, rectus abdominis; TA, transversus abdominis; OA, oblique abdominals. ACM, abdominal core muscles at L3; NAMA, normal attenuation muscle area; TAMA, total abdominal muscle area; BMI, body mass index, NAMA/TAMA index, normal attenuation muscle area/total abdominal muscle area×100.

Overall, in distinguishing all CAC from NCs with T2DM, NAMA/BMI showed a higher AUC than TAMA, NAMA and NAMA/TAMA index in most muscles (6 out of 7), except for ES (**Figure 4a****).** The range of AUC-value in NAMA/BMI, NAMA/TAMA index, NAMA and TAMA was 0.919-0.950, 0.765-0.950, 0.779-0.867 and 0.528-0.716, respectively. The range of sensitivity in NAMA/BMI, NAMA/TAMA index, NAMA and TAMA was 66.15%-90.77%, 70.77%-92.31%, 49.23%-80.00%, and 13.85%-84.62%, respectively. The range of specificity in NAMA/BMI, NAMA/TAMA index, NAMA, and TAMA was 69.77%-95.35%, 74.42%-93.02%, 74.43%-99.99%, and 41.86%-99.99%, respectively. In distinguishing three CAC subgroups (mild, moderate, or severe) from NCs, NAMA/BMI and NAMA/TAMA index achieved higher AUC than TAMA and NAMA in all muscles (**Figures 4b-d**).

**Figure 5:**
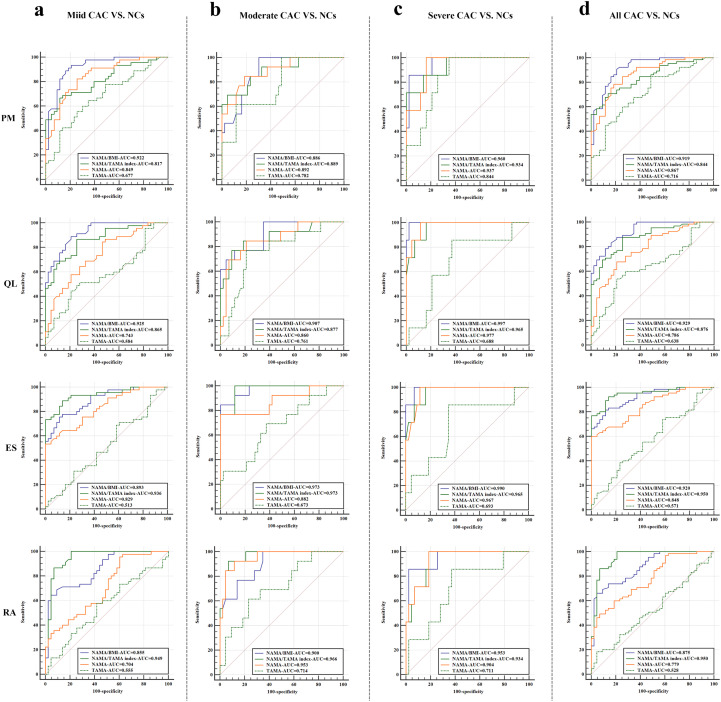
Receiver operating characteristic (ROC) curves for four DSCT measurements (TAMA, NAMA, NAMA/BMI, and NAMA/TAMA index) in four representative muscles to differentiate T2DM patients with mild, moderate, or severe CAC and negative controls (NCs) **(a-c)**, as well as to distinguish between all CAC from NCs **(d)**. PM, psoas major; QL, quadratus lumborum; ES, erector spinae; RA, rectus abdominis; NAMA, normal attenuation muscle area; TAMA, total abdominal muscle area; BMI, body mass index, NAMA/TAMA index, normal attenuation muscle area/total abdominal muscle area×100; AUC, area under the curve.

**Figure 5** shows the ROC curves used to identify the optimal DSCT measurement biomarkers in representative muscles (PM, QL, ES, and RA) to differentiate T2DM patients with CAC subgroup (mild, moderate, or severe) and NCs, as well as to distinguish between all CAC subjects from NCs.

### 3.4 Correlation analysis between muscle parameters and CACS

**Figure 6:**
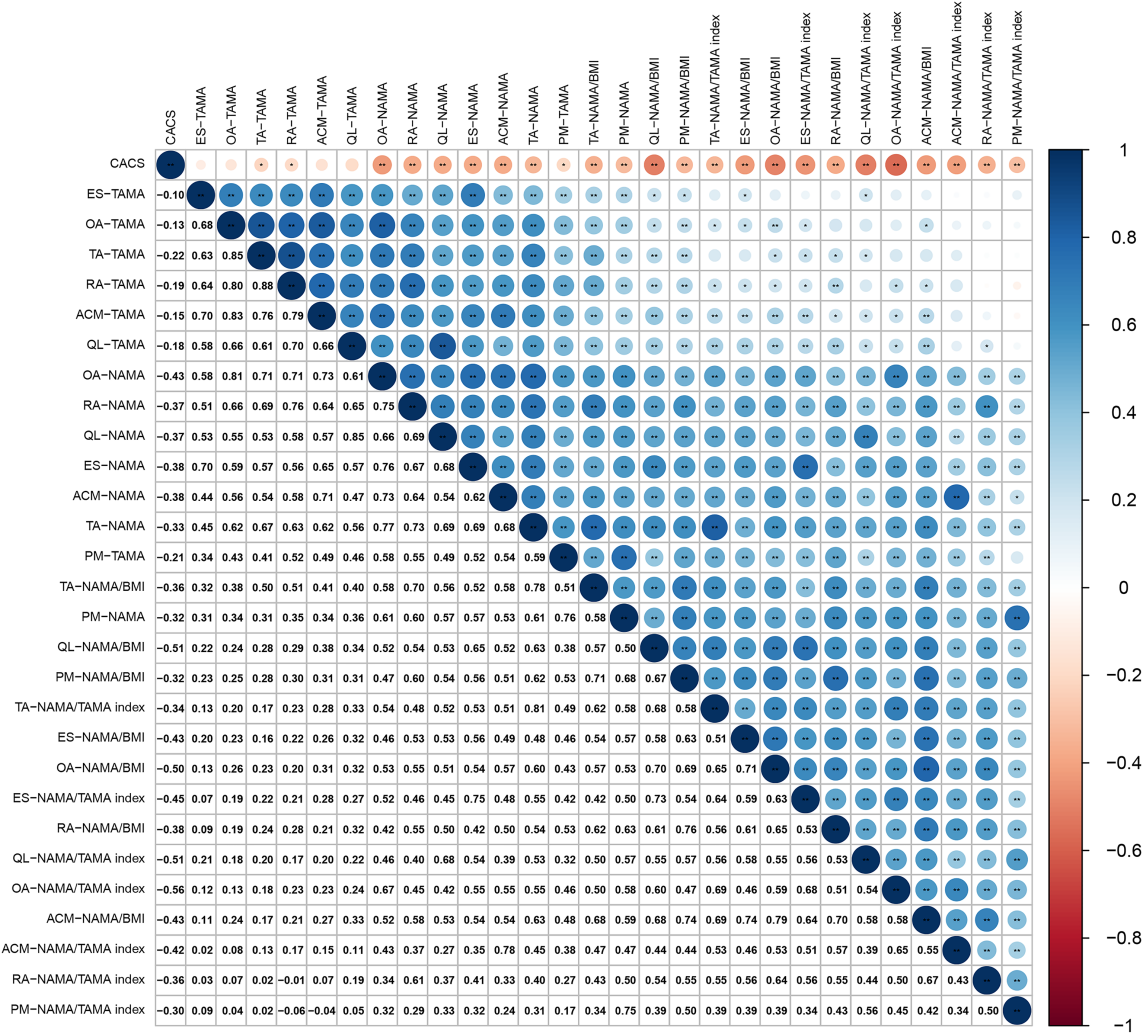
Heat-map displaying the correlation coefficient of DSCT measurements and CACS. Statistically significant, *P < 0.05, **P < 0.01, ***P < 0.001. Different sizes of the blue and red dots: the larger the dot size, the greater the absolute value of the correlation coefficient. CACS, coronary artery calcification score; PM, psoas major; QL, quadratus lumborum; ES, erector spinae; RA, rectus abdominis; TA, transversus abdominis; OA, oblique abdominals; ACM, abdominal core muscles at L3; NAMA, normal attenuation muscle area; TAMA, total abdominal muscle area; BMI, body mass index, NAMA/TAMA index, normal attenuation muscle area/total abdominal muscle area×100.

**Figure 6** shows the correlation coefficient between 28 DSCT-derived muscle measurements and CACS in T2DM patients. The heat-map revealed that in addition to four muscle parameters (ACM -TAMA, QL -TAMA, OA -TAMA, and ES -TAMA), the remaining 24 muscle parameters exhibited negative correlations with CACS significantly (r =-0.13 to -0.56, all *P* < 0.01). Among which, NAMA/TAMA index in OA exhibited the strongest negative correlation with the CACS (r=-0.56, *P* < 0.01).

### 3.5 Risk factors for positive CAC

**Figure 7:**
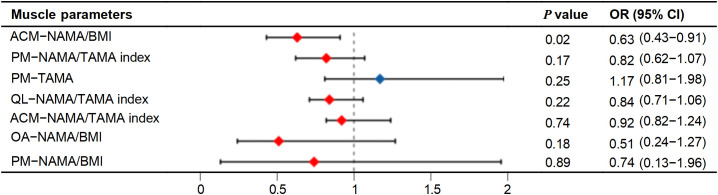
Forest plot depicting odds ratios of muscle parameters for CAC positive patients with T2DM. CAC, coronary artery calcification; PM, psoas major; QL, quadratus lumborum; OA, oblique abdominals; ACM, abdominal core muscles at L3. NAMA, normal attenuation muscle area; TAMA, total abdominal muscle area; BMI, body mass index, NAMA/TAMA index, normal attenuation muscle area/total abdominal muscle area×100.

Given that the TAMA, NAMA/BMI, NAMA/TAMA index, and NAMA (across all seven muscles, with TAMA excluded for the ACM, OA, QL, and RA) were significantly correlated with CAC in patients with T2DM, these indices were included as factors in a univariate logistic regression analysis (**Table 3****).** The TAMA in the PM, along with the NAMA/BMI, NAMA/TAMA index, and NAMA across all seven muscles, were identified as factors associated with CAC in the univariate logistic regression analysis (*P* < 0.05). Subsequently, multivariate logistic regression analysis showed that NAMA/BMI in the ACM (odds ratio=0.63) was an independent risk factor for positive CAC (*P* < 0.05) (**Figure 7****).**

**Table 3:** Independent risk factors for CAC positive patients with T2DM.

Variables	Univariate analysis		Multivariate analysis^&^		VIF
	Odds ratio (95%CI)	*P*	Odds ratio (95%CI)	*P*	
ACM-NAMA/BMI	0.67 (0.56-0.79)	<0.01	0.63 (0.43-0.91)	0.02	5.39
PM-NAMA/TAMA index	0.87 (0.83-0.93)	<0.01	0.82 (0.62-1.07)	0.17	2.21
PM-TAMA	0.85 (0.77-0.92)	<0.01	1.17 (0.81-1.98)	0.25	2.28
QL-NAMA/TAMA index	0.84 (0.79-0.90)	<0.01	0.84 (0.71-1.06)	0.22	2.36
ACM-NAMA/TAMA index	0.92 (0.88-0.95)	<0.01	0.92 (0.82-1.24)	0.74	2.27
OA-NAMA/BMI	0.52 (0.40-0.66)	<0.01	0.51 (0.24-1.27)	0.18	4.53
PM-NAMA/BMI	0.17 (0.08-0.34)	<0.01	0.74 (0.13-1.96)	0.89	5.31
OA-NAMA	0.90 (0.86-0.95)	<0.01	–	–	104.1
ES-NAMA	0.84 (0.78-0.89)	<0.01	–	–	94.7
TA-NAMA	0.52 (0.39-0.67)	<0.01	–	–	43.7
RA-NAMA	0.80 (0.72-0.88)	<0.01	–	–	67.0
QL-NAMA	0.63 (0.51-0.77)	<0.01	–	–	57.7
ACM-NAMA	0.96 (0.95-0.98)	<0.01	–	–	81.5
PM-NAMA	0.70 (0.61-0.80)	<0.01	–	–	57.9
OA-NAMA/TAMA index	0.87 (0.82-0.92)	<0.01	–	–	46.2
ES-NAMA/TAMA index	0.77 (0.70-0.85)	<0.01	–	–	53.4
RA-NAMA/TAMA index	0.75 (0.67-0.85)	<0.01	–	–	30.0
TA-NAMA/TAMA index	0.85 (0.80-0.91)	<0.01	–	–	27.5
ES-NAMA/BMI	0.56 (0.45-0.69)	<0.01	–	–	32.3
RA-NAMA/BMI	0.35 (0.24-0.53)	<0.01	–	–	42.8
QL-NAMA/BMI	0.05 (0.02-0.17)	<0.01	–	–	43.5
TA-NAMA/BMI	0.25 (0.14-0.45)	<0.01	–	–	50.9
TA-TAMA	0.95 (0.84-1.07)	0.38	–	–	23.3
RA-TAMA	0.99 (0.94-1.04)	0.58	–	–	53.1

CI, confidence interval; PM, psoas major; QL, quadratus lumborum; ES, erector spinae; RA, rectus abdominis; TA, transversus abdominis; OA, oblique abdominals; ACM, abdominal core muscles at L3. NAMA, normal attenuation muscle area; TAMA, total abdominal muscle area; BMI, body mass index, NAMA/TAMA index, normal attenuation muscle area/total abdominal muscle area×100; VIF, variance inflation factor; CAC, coronary artery calcification. **^&^**, In addition to muscle parameters, the multivariable logistic regression analysis was adjusted for age, sex, LDL-C, HDL-C, TG, HbA1c (%), diabetes duration (months), family history of CVD (n, %), osteoporosis (n, %), hypertension (n, %), alcohol history (n, %), and smoking history (n, %).

## 4 Discussion

This study utilized DSCT to investigate the relationship between abdominal muscle mass and CAC in patients with T2DM. Our primary findings suggest that NAMA/BMI demonstrate effective diagnostic capabilities for positive CAC patients across different degrees of severity. In addition, we found significant correlations between CACS and DSCT muscle parameters. Importantly, NAMA/BMI derived from ACM served as an independent risk factor for the positive CAC.

As anticipated, we found positive CAC patients with T2DM exhibited markedly lower muscle cross-sectional area and related derived indices compared with NCs. Consistent with earlier evidence, these findings demonstrate that abdominal muscle infiltration associated with chronic diseases (e.g., uremia, nephrotic syndrome, and hypertension) may contribute to the development of atherosclerosis (8, 13, 16–18). The observed alterations in muscle parameters likely reflect a concurrent depletion and degeneration of muscle tissue during the progression of T2DM-associated CAC (8, 12, 19). This association may be explained through two main mechanisms. On the one hand, in T2DM patients, greater CAC severity—compared to that in CAC-negative individuals—correlated with hyperglycemia and a chronic inflammatory state. These conditions suppress muscle protein synthesis, activate atrophy-related catabolic pathways, and promote ectopic fat deposition within muscle, collectively impairing both muscle mass and quality (20–22). On the other hand, increasing CAC severity exacerbated insulin resistance, which further disrupted protein metabolism and intensified lipolysis (23–25). This pathophysiological cascade aggravated muscle loss and fat infiltration, thereby establishing a direct link between deteriorating muscle integrity and advancing vascular injury.

Overall, in the diagnosis of CAC among patients with T2DM, the NAMA/BMI index performed better than NAMA alone or the NAMA/TAMA index. Given that muscle cross-sectional area reflects absolute muscle quantity but does not account for individual differences in body size, in taller or larger individuals, it may overestimate true muscle reserves. In contrast to TAMA, which reflects overall muscle quantity, NAMA represents the area of healthy, non-infiltrated muscle tissue, while its counterpart LAMA directly quantifies intramuscular fat infiltration. Therefore, NAMA and NAMA-derived indices are regarded as imaging markers of “muscle quality” with higher values indicating less fatty infiltration and better metabolic health. The lack of significant association between LAMA and CAC may be attributable to two factors: first, LAMA represents a composite measure that includes both IMAT and intramyocellular lipids, but these compartments may have differing metabolic implications. While IMAT has been consistently associated with insulin resistance and inflammation, intramyocellular lipids themselves can exist in metabolically inert storage forms that do not directly promote atherosclerosis (26, 27); second, the lack of significant association between LAMA and CAC in our study may therefore reflect this heterogeneity within the LAMA compartment (28). However, when used in isolation, its measurements are susceptible to various influences, including CT acquisition parameters, metabolic comorbidities, and hydration status, which compromise its stability and diagnostic performance. Although the NAMA/TAMA index incorporates the relative relationship between muscle quality and quantity to some extent, it still does not eliminate the confounding effect of individual body size; in particular, when total muscle area itself is strongly influenced by body size, the ratio may also be biased (29, 30). By comparison, normalizing NAMA by BMI effectively reduces body-size bias, substantially attenuates interference from body-size factors, and more accurately reflects intrinsic muscle reserves and functional status. This approach compensates for the limitation of relying solely on cross-sectional area to assess functional capacity, thereby improving both the accuracy and stability of diagnosis.

In this study, we found that assessment of abdominal muscle mass exhibited good diagnostic performance for CAC in patients with T2DM, and in particular, we found that NAMA/BMI derived from ACM assessment achieved the best overall diagnostic performance (AUC = 0.997). This finding is similar to the results reported by Kim et al. (31), who used composite abdominal muscle indices to evaluate cardiovascular risk associated with aortosclerosis resulting from chronic kidney disease. Critically, inter- and intramuscular fat deposition contributes to reduced insulin sensitivity and increased cardiovascular mortality risk (12, 29). Mechanistically, the elevated lipid content within muscle cells upregulates genes for oxidative metabolism (11, 32). This pathway suggests that more severe abdominal fat infiltration could promote CAC progression, thereby linking ectopic fat deposition directly to vascular pathology (12, 33, 34). Notably, NAMA/BMI can more accurately reflect the internal structure of muscle and the degree of fat infiltration, providing imaging evidence for the “muscle–vascular axis” theory and suggesting that muscle quality and vascular health may share common pathophysiological mechanisms (22, 25). Furthermore, we found that NAMA/BMI derived from ACM assessment was an independent predictor of CAC. By encompassing multiple abdominal muscle groups, ACM serves as a composite index that more comprehensively reflects whole-body muscle quality, thereby conferring upon this metric robust diagnostic performance and predictive value for CAC.

Interestingly, this study employed DSCT to simultaneously assess CACS and abdominal muscle quality, uncovering a potential link between them. DSCT offers unique value in CAC evaluation, enabling accurate coronary calcification quantification with high spatial resolution and minimal added radiation in routine imaging. Its “one−stop” design integrates vascular and musculoskeletal evaluations without extra contrast or radiation, making it suitable for T2DM and promising for other metabolic diseases where muscle-vascular interactions drive progression and risk stratification (35).

This study has several limitations. First, there were imbalances in sample size across groups, and the overall sample size was relatively small, particularly in the high-CACS subgroup, which may increase the risk of Type II errors. Second, longitudinal studies are needed to validate our findings and further confirm the temporal relationship between muscle composition changes and CAC progression. Third, despite the adjustment for a variety of potential confounders, residual confounding may still exist. Finally, although the use of non-gated CT inevitably introduces artifacts, it correlates well with gated CT for CAC scoring, supporting its utility in risk stratification.

## 5 Conclusion

Abdominal muscle mass measured by DSCT was significantly associated with CACS in T2DM patients. Abdominal muscle parameters demonstrate effective diagnostic capabilities for CAC across different degrees of severity, with NAMA/BMI emerging as the preferred CT biomarker. In addition, NAMA/BMI in the ACM may serve as a potential biomarker for prediction of positive CAC.

## Data Availability

The original contributions presented in the study are included in the article/**Supplementary Material**. Further inquiries can be directed to the corresponding author.

## References

[1] Strain WD Paldánius PM . Diabetes, cardiovascular disease and the microcirculation. Cardiovasc Diabetol. (2018) 17:57. doi: 10.1186/s12933-018-0703-2, PMID: 29669543 PMC5905152

[2] Bonnefond A Florez JC Loos RJF Froguel P . Dissection of type 2 diabetes: A genetic perspective. Lancet Diabetes Endocrinol. (2025) 13:149–64. doi: 10.1016/S2213-8587(24)00339-5, PMID: 39818223

[3] Chao C-T Han D-S . Operationalizing vascular frailty: Structural and functional vascular ageing as determinants of geriatric phenotypes. J Formosan Med Assoc. (2025) 86(9):1094–134. doi: 10.1016/j.jfma.2025.10.042, PMID: 41188125

[4] Wadhera RK Dhruva SS Bikdeli B Bonaca MP Kittleson MM Ko DT . Cardiovascular statistics in the United States, 2026. JACC. (2026) 87(9):1094–134. doi: 10.1016/j.jacc.2025.12.027. S0735109725105585. PMID: 41524687

[5] Foraker R Sperling L Bratzke L Budoff M Leppert M Razavi AC . Opportunistic detection of coronary artery calcium on noncardiac chest computed tomography: An emerging tool for cardiovascular disease prevention: a scientific statement from the American heart association. Circulation. (2025) 152(19):e391-e401. doi: 10.1161/CIR.0000000000001382, PMID: 41099128 PMC13034728

[6] Han D Tzolos E Park R Gransar H Hyun M Friedman JD . Effects of evolocumab on coronary plaque composition and microcalcification activity by coronary PET and CT angiography. JACC: Cardiovasc Imaging. (2025) 18:589–99. doi: 10.1016/j.jcmg.2025.01.005, PMID: 40178463 PMC12058403

[7] Yakubov SJ Van Mieghem NM Oh JK Ito S Grubb KJ O’Hair D . Impact of transcatheter or surgical aortic valve performance on 5-year outcomes in patients at ≥ intermediate risk. J Am Coll Cardiol. (2025) 85:1419–30. doi: 10.1016/j.jacc.2025.02.009, PMID: 40175015

[8] Morel A Ouamri Y Ségaux L Zaidan L Moryoussef M Mulé S . Myosteatosis as a new risk factor of surgical complications in kidney transplant recipients: A retrospective study. J Cachexia Sarcopenia Muscle. (2025) 16:e13746. doi: 10.1002/jcsm.13746, PMID: 40304205 PMC12041939

[9] Lan Z Liang Q Li L Liu F Chen A Ye Y . TRIM16 mediates K63-linked ubiquitination of DAB2 to facilitate vascular calcification. Circ Res. (2025) 137:551–68. doi: 10.1161/CIRCRESAHA.125.326520, PMID: 40575853

[10] Erley J Breckow J Roedl K Ozga A-K Duoerkongjiang A De Heer G . Dual-energy CT liver fat fraction as prognostic imaging biomarker in critically ill patients. Eur Radiol. (2025) 36(2):1341–50. doi: 10.1007/s00330-025-11851-3, PMID: 40770141 PMC12953330

[11] Erley J Roedl K Ozga A-K De Heer G Schubert N Breckow J . Dual-energy CT muscle fat fraction as a new imaging biomarker of body composition and survival predictor in critically ill patients. Eur Radiol. (2024) 34:7408–18. doi: 10.1007/s00330-024-10779-4, PMID: 38777903 PMC11519288

[12] Liu F-P Guo M-J Yang Q Li Y-Y Wang Y-G Zhang M . Myosteatosis is associated with coronary artery calcification in patients with type 2 diabetes. World J Diabetes. (2024) 15:429–39. doi: 10.4239/wjd.v15.i3.429, PMID: 38591084 PMC10999038

[13] Sachdev PS Bentvelzen AC Gustafson D Hansra GK Hosoki S Jiang J . Vascular cognitive impairment and dementia. JACC. (2026) 87:52–76. doi: 10.1016/j.jacc.2025.11.008, PMID: 41498479 PMC13553213

[14] Adler AI Coleman RL Leal J Whiteley WN Clarke P Holman RR . Post-trial monitoring of a randomised controlled trial of intensive glycaemic control in type 2 diabetes extended from 10 years to 24 years (UKPDS 91). Lancet. (2024) 404:145–55. doi: 10.1016/S0140-6736(24)00537-3, PMID: 38772405

[15] Holman RR Paul SK Bethel MA Matthews DR Neil HAW . 10-year follow-up of intensive glucose control in type 2 diabetes. N Engl J Med. (2008) 359:1577–89. doi: 10.1056/NEJMoa0806470, PMID: 18784090

[16] Chen Q Xiao H Li Y Jian L Zhang L Lai B . A transformer-based prognostic signature integrating tumor and body composition CT images predicts postoperative recurrence in gastric cancer. NPJ Digit Med. (2025) 9(1):12. doi: 10.1038/s41746-025-02183-z, PMID: 41339473 PMC12775138

[17] Jung HN Cho YK Kim HS Kim EH Lee MJ Lee WJ . Association between hypertension and myosteatosis evaluated by abdominal computed tomography. Hypertens Res. (2023) 46:845–55. doi: 10.1038/s41440-022-01157-y, PMID: 36635524

[18] Kim S Choi G Song Y Yoon H Jeong HM Gu JE . Low muscle mass in patients receiving hemodialysis: Correlations with vascular calcification and vascular access failure. JCM. (2021) 10:3698. doi: 10.3390/jcm10163698, PMID: 34441991 PMC8396811

[19] Jensky NE Criqui MH Wright CM Wassel CL Alcaraz JE Allison MA . The association between abdominal body composition and vascular calcification. Obesity. (2011) 19:2418–24. doi: 10.1038/oby.2011.70, PMID: 21475146

[20] Dzaye O Razavi AC Dardari ZA Wang FM Honda Y Nasir K . Polygenic risk scores and extreme coronary artery calcium phenotypes (CAC = 0 and CAC≥1000) in adults ≥75 years old: The ARIC study. Circ: Cardiovasc Imaging. (2024) 17(11):e016377. doi: 10.1161/CIRCIMAGING.123.016377, PMID: 39534973 PMC11576240

[21] Cameron NA Petito LC Colangelo LA Gunderson EP Catov JM Grobman WA . Prepregnancy cardiovascular health, gestational diabetes, and coronary artery calcium. JAMA Cardiol. (2025) 10:888. doi: 10.1001/jamacardio.2025.1887, PMID: 40560557 PMC12199180

[22] Tian L Jaeger BC Scialla JJ Budoff MJ Mehta RC Jaar BG . Progression of coronary artery calcification and risk of clinical events in CKD: The chronic renal insufficiency cohort study. Am J Kidney Dis. (2025) 85:67–77.e1. doi: 10.1053/j.ajkd.2024.06.018, PMID: 39154888 PMC12278985

[23] Meer R Hoek AG Bouman EJ Doesburg T Elders PJM De Jong PA . Association between lower extremity arterial calcification and coronary arterial calcification in a population at increased risk of cardiovascular disease. BMJ Open Diabetes Res Care. (2024) 12:e003811. doi: 10.1136/bmjdrc-2023-003811, PMID: 38336383 PMC10859972

[24] Meer R Oughzou I Hoek AG Dal Canto E Blom MT Doesburg T . The association between peripheral medial and intimal arterial calcification patterns with central arterial stiffness in individuals with type 2 diabetes mellitus: The cross-sectional early-HFpEF study. J Cardiovasc Computed Tomography. (2025) 19:444–52. doi: 10.1016/j.jcct.2025.04.005, PMID: 40300916

[25] Gerber Y Gabriel KP Jacobs DR Liu JY Rana JS Sternfeld B . The relationship of cardiorespiratory fitness, physical activity, and coronary artery calcification to cardiovascular disease events in CARDIA participants. Eur J Prev Cardiol. (2025) 32:52–62. doi: 10.1093/eurjpc/zwae272, PMID: 39158112 PMC11700624

[26] Mezincescu AM Rudd A Cheyne L Horgan G Philip S Cameron D . Comparison of intramyocellular lipid metabolism in patients with diabetes and male athletes. Nat Commun. (2024) 15:3690. doi: 10.1038/s41467-024-47843-y, PMID: 38750012 PMC11096352

[27] Pesta D Anadol-Schmitz E Sarabhai T Op Den Kamp Y Gancheva S Trinks N . Determinants of increased muscle insulin sensitivity of exercise-trained versus sedentary normal weight and overweight individuals. Sci Adv. (2025) 11:eadr8849. doi: 10.1126/sciadv.adr8849, PMID: 39742483 PMC11691647

[28] Li C Yu K Shyh-Chang N Jiang Z Liu T Ma S . Pathogenesis of sarcopenia and the relationship with fat mass: Descriptive review. J Cachexia Sarcopenia Muscle. (2022) 13:781–94. doi: 10.1002/jcsm.12901, PMID: 35106971 PMC8977978

[29] Boshnjaku A Krasniqi E . Diagnosing sarcopenia in clinical practice: international guidelines vs. population-specific cutoff criteria. Front Med. (2024) 11:1405438. doi: 10.3389/fmed.2024.1405438, PMID: 39131085 PMC11310033

[30] Dong X Li N Zhu C Wang Y Shi K Pan H . Diagnosis of coronary artery disease in patients with type 2 diabetes mellitus based on computed tomography and pericoronary adipose tissue radiomics: a retrospective cross-sectional study. Cardiovasc Diabetol. (2023) 22:14. doi: 10.1186/s12933-023-01748-0, PMID: 36691047 PMC9869509

[31] Kim A Lee C Kang B-K Kim M Choi JW . Myosteatosis and aortic calcium score on abdominal CT as prognostic markers in non-dialysis chronic kidney disease patients. Sci Rep. (2024) 14:7718. doi: 10.1038/s41598-024-58293-3, PMID: 38565556 PMC10987640

[32] Lipina C Hundal HS . Lipid modulation of skeletal muscle mass and function. J Cachexia Sarcopenia Muscle. (2017) 8:190–201. doi: 10.1002/jcsm.12144, PMID: 27897400 PMC5377414

[33] Masi S Rye M Roussac A Naghdi N Rosenstein B Bailey JF . Comparison of paraspinal muscle composition measurements using IDEAL fat-water and T2-weighted MR images. BMC Med Imaging. (2023) 23:48. doi: 10.1186/s12880-023-00992-w, PMID: 36997912 PMC10064674

[34] Jung HN Cho YK Kim HS Kim EH Lee MJ Park J-Y . Association of serum gamma-glutamyl transferase with myosteatosis assessed by muscle quality mapping using abdominal computed tomography. Clin Imaging. (2023) 93:4–11. doi: 10.1016/j.clinimag.2022.10.009, PMID: 36335677

[35] Luo J Wang Q Liu W Liao H Qing W Zhang M . Computed tomography provides a “one-stop-shop” targeted analysis for coronary artery calcification and osteoporosis: A review. Front Endocrinol. (2025) 16:1356831. doi: 10.3389/fendo.2025.1356831, PMID: 40093749 PMC11906312

