# Supplementary material for: Association between abdominal muscle mass measured by dual-source computed tomography and coronary artery calcification in patients with Type 2 diabetes mellitus
Source: Front Endocrinol (Lausanne). 2026 Mar 26;17:1803735. doi: 10.3389/fendo.2026.1803735 (PMC13061661; doi:10.3389/fendo.2026.1803735)
Supplement: Supplementary file 1 [file Table1.docx]

Supplementary Table 1. DSCT measurements of individual muscles among NCs CAC, Mild CAC, Moderate CAC, and Severe CAC participants.

| Muscle |  | IMAT (cm^2^) | | | | |  |  | LAMA (cm^2^) | | | | |  |  | FF (%) | | | | |  |  | LAMA/BMI (cm^2^/kg/m^2^) | | | | |  |  |
| --- | --- | --- | --- | --- | --- | --- | --- | --- | --- | --- | --- | --- | --- | --- | --- | --- | --- | --- | --- | --- | --- | --- | --- | --- | --- | --- | --- | --- | --- |
|  |  | NCs  CAC  (n=43) | Mild  CAC  (n=45) | Moderate CAC  (n=13) | Severe CAC  (n=7) | All  CAC  (n=65) | *P*  for trend |  | NCs  CAC  (n=43) | Mild  CAC  (n=45) | Moderate CAC  (n=13) | Severe CAC  (n=7) | All  CAC  (n=65) | *P*  for trend |  | NCs  CAC  (n=43) | Mild  CAC  (n=45) | Moderate CAC  (n=13) | Severe CAC  (n=7) | All  CAC  (n=65) | *P*  for trend |  | NCs  CAC  (n=43) | Mild  CAC  (n=45) | Moderate CAC  (n=13) | Severe CAC  (n=7) | All  CAC  (n=65) | *P*  for trend |  |
| PM |  | 0.65±0.07 | 0.67±0.08 | 0.70±0.14 | 0.71±0.16 | 0.68±0.10 | <0.05 |  | 4.23±0.24 | 4.32±0.24 | 3.55±0.43 | 4.30±0.68 | 4.16±0.46 | <0.05 |  | 12.84±0.98 | 12.27±0.79 | 15.11±1.64 | 14.81±2.34 | 13.11±1.75 | <0.05 |  | 1.72±0.08 | 1.75±0.08 | 1.59±0.18 | 1.93±0.28 | 1.74±0.16 | <0.05 |  |
| QL |  | 0.31±0.04 | 0.34±0.03 | 0.33±0.04 | 0.32±0.08 | 0.34±0.04 | <0.05 |  | 2.41±0.15 | 2.30±0.12 | 2.18±0.28 | 2.13±0.33 | 2.26±0.20 | <0.05 |  | 11.68±0.96 | 12.67±0.88 | 14.22±1.65 | 13.57±2.76 | 13.08±1.47 | <0.05 |  | 0.99±0.06 | 0.94±0.04 | 0.97±0.12 | 0.94±0.12 | 0.95±0.07 | <0.05 |  |
| ES |  | 2.46±0.24 | 15.11±1.64 | 15.11±1.64 | 15.11±1.64 | 15.11±1.61 | <0.05 |  | 12.52±0.70 | 12.87±0.62 | 12.97±1.23 | 16.02±1.43 | 13.23±1.30 | <0.05 |  | 13.21±1.11 | 14.08±1.12 | 15.34±1.58 | 15.72±2.22 | 14.51±1.49 | <0.05 |  | 5.18±0.28 | 5.37±0.29 | 5.85±0.54 | 7.11±0.50 | 5.65±0.66 | <0.05 |  |
| RA |  | 1.19±0.18 | 1.34±0.21 | 1.09±0.35 | 1.36±0.46 | 1.29±0.29 | <0.05 |  | 9.75±0.49 | 10.03±0.56 | 7.59±0.76 | 7.06±0.96 | 9.22±1.39 | <0.05 |  | 9.14±0.57 | 9.62±0.49 | 7.18±0.93 | 7.44±1.16 | 8.90±1.29 | <0.05 |  | 4.04±0.19 | 4.08±0.20 | 3.41±0.32 | 3.20±0.43 | 3.85±0.43 | <0.05 |  |
| TA |  | 1.14±0.17 | 1.18±0.18 | 1.33±0.33 | 1.29±0.43 | 1.22±0.25 | <0.05 |  | 3.47±0.21 | 3.26±0.21 | 3.52±0.29 | 3.31±0.55 | 3.32±0.29 | <0.05 |  | 23.95±2.19 | 23.16±2.25 | 31.90±4.27 | 29.93±7.19 | 25.64±5.10 | <0.05 |  | 1.44±0.09 | 1.34±0.08 | 1.58±0.12 | 1.47±0.28 | 1.40±0.16 | <0.05 |  |
| OA |  | 1.98±0.19 | 1.82±0.13 | 2.17±2.30 | 1.60±0.28 | 1.87±1.02 | <0.05 |  | 14.80±0.96 | 13.68±0.89 | 14.31±1.47 | 15.75±2.07 | 14.03±1.33 | <0.05 |  | 15.69±1.18 | 14.89±0.98 | 19.66±2.08 | 16.28±2.72 | 15.99±2.40 | <0.05 |  | 6.13±0.39 | 5.57±0.34 | 6.34±0.54 | 6.92±0.86 | 5.87±0.66 | <0.05 |  |
| ACM |  | 10.76±0.70 | 10.74±0.73 | 10.57±0.95 | 10.17±1.63 | 10.64±0.90 | <0.05 |  | 39.07±2.01 | 36.98±1.68 | 37.90±2.92 | 43.62±4.03 | 37.88±3.04 | <0.05 |  | 16.91±1.15 | 16.76±1.07 | 20.11±1.93 | 18.89±2.56 | 17.66±2.01 | <0.05 |  | 16.07±0.73 | 15.16±0.64 | 16.97±1.14 | 19.35±1.59 | 15.97±1.64 | <0.05 |  |

Note: Data represent mean± SD. An adjustment in statistical significance thresholds has been implemented by using Benjamini-Hochberg correction.

Abbreviations: CAC=coronary artery calcification, NCs CAC = CAC Negative Controls, PM=psoas major, QL = quadratus lumborum, ES= erector spinae, RA =rectus abdominis, TA=transversus abdominis, OA = oblique abdominals, ACM =abdominal core muscles at L3.

FF = fat fraction, NAMA = normal attenuation muscle area, IMAT= intermuscular adipose tissue, LAMA= low attenuation muscle area, TAMA= total abdominal muscle area, BMI= body mass index, NAMA/TAMA index= normal attenuation muscle area/total abdominal muscle area×100.
